# Impact of new‐onset and preexisting neurological disorders in COVID‐19 patients

**DOI:** 10.1002/brb3.3066

**Published:** 2023-05-18

**Authors:** Sarah Lindemann, Markus Böhm, Falk Gonnert, Julian Bösel, Roger Schubert, Rita Musleh, Albrecht Günther

**Affiliations:** ^1^ Department of Neurology SRH Waldklinikum Gera Gera Germany; ^2^ Institute of Medical Statistics, Computer and Data Sciences Jena University Hospital Jena Germany; ^3^ Department of Anesthesiology and Intensive Care Medicine SRH Waldklinikum Gera Gera Germany; ^4^ Department of Neurology Klinikum Kassel Kassel Germany; ^5^ Department of Neurology University Hospital Heidelberg Heidelberg Germany; ^6^ Department of Neurology Jena University Hospital Jena Germany

**Keywords:** acute neurological complications, COVID‐19, hospital mortality, neurological diseases, SARS‐CoV2

## Abstract

**Background and Purpose:**

Coronavirus disease (COVID‐19) is still considered a global pandemic. The prognosis of COVID‐19 patients varies greatly. We aimed to assess the impact of preexisting, chronic neurological diseases (CNDs) and new‐onset acute neurological complications (ANCs) on the disease course, its complications, and outcomes.

**Methods:**

We conducted a monocentric retrospective analysis from all hospitalized COVID‐19 patients between May 1, 2020 and January 31, 2021. Employing multivariable logistic regression models, we explored the association of CNDs and ANCs separately with hospital mortality and functional outcome.

**Results:**

A total of 250 among 709 patients with COVID‐19 had CNDs. We found a 2.0 times higher chance of death (95% confidence interval [CI]: 1.37–2.92) for CND patients than for non‐CND patients. The chance for an unfavorable functional outcome (modified Rankin Scale > 3 at discharge) was 1.67 times higher in patients with CNDs than those without (95% CI: 1.07–2.59). Furthermore, 117 of all patients had 135 ANCs in total. We observed a 1.86 times higher chance to die (95% CI: 1.18–2.93) for patients with ANCs than without. The chance for a worse functional outcome was 3.6‐fold higher in ANC patients than without (95% CI: 2.22–6.01). Patients with CNDs had 1.73 times higher odds for developing ANCs (95% CI: 0.97–3.08).

**Conclusion:**

Preexisting neurologic disorders or ANCs in COVID‐19 patients were associated with higher mortality and poorer functional outcome at discharge. Furthermore, development of acute neurologic complications was more frequent in patients with preexisting neurologic disease. Early neurological evaluation appears to be an important prognostic factor in COVID‐19 patients.

## Graphical abstract

Preexisting neurologic disorders or acute neurological complications in COVID‐19 patients were associated with higher mortality and poorer functional outcome at discharge. Furthermore, development of acute neurologic complications was more frequent in patients with preexisting neurologic disease. Early neurological evaluation appears to be an important prognostic factor in COVID‐19 patients.

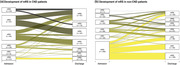

## 1 INTRODUCTION

At the end of 2019, a highly transmissible novel coronavirus, designated as SARS‐CoV2 (severe acute respiratory syndrome corona virus type 2), was discovered to be the pathogen causing the new worldwide pandemic, subsequently named COVID‐19 (coronavirus disease 2019) (Hu et al., 2021).

The clinical course of this infection is highly variable, ranging from being asymptomatic or with mild respiratory symptoms to multiple organ failure and even death (Al‐Tawfiq et al., 2020). Therefore, mortality rates among COVID‐19 patients vary greatly (Zhou et al., 2020). Researchers worldwide attempt to identify possible risk factors for a severe course of infection. This is considered important for predicting the hospitalization as well as the intensive care unit (ICU) admission need. Age, male sex, cardiovascular risk factors such as hypertension, diabetes, and obesity as well as chronic respiratory disease were identified as risk factors for severe/fatal COVID‐19 (Zhou et al., 2020). Recent studies showed an association between preexisting cerebrovascular diseases and a severe disease course as well as higher mortality in COVID‐19 patients (Aggarwal et al., 2020; Pranata et al., 2020).

Moreover, COVID‐19 was shown to be associated with various acute neurological complications (ANCs). Multiple studies described COVID‐19‐associated cerebrovascular complications and inflammatory syndromes of the central and peripheral nervous systems as well as encephalopathy and miscellaneous disorders (Leven & Bösel, 2021). The incidence of stroke among COVID‐19 patients varies between 1.4% (Nannoni et al., 2021) and 5.9% (Frontera, Sabadia, et al., 2021; Travi et al., 2021). The incidence of encephalopathic complications ranged between 2.8% (Rifino et al., 2021) and 31.8% (Liotta et al., 2020). In addition, a high number of neurological symptoms such as headache, dizziness, myalgias, anosmia, and dysgeusia were reported (Maury et al., 2021).

In this study, we aimed to investigate the role of preexisting chronic neurological diseases (CNDs) as a potential risk factor for a more severe course or fatal outcome of SARS‐CoV2 infection. In addition, we aimed to detect the association of such preexisting diseases with ANCs and their impact on the clinical course of COVID‐19 disease.

## 2 METHODS

### 2.1 Patient selection and characteristics

All patients older than 18 years admitted to a regional German secondary hospital (SRH Waldklinikum Gera) between May 1, 2020 and January 31, 2021 with a positive polymerase chain reaction (PCR) test for SARS‐CoV2 from a combined nose and throat swab were included in this monocentric retrospective analysis. Patients in psychiatry were excluded for medical records not being available. Patients admitted to hospital for another reason than SARS‐CoV2 only were not included if they developed no symptoms and were soon released. Patients who were infected in hospital or developed symptoms on a later date were included.

The study protocol was approved by the Ethics committee of the State Chamber for Physicians of Thuringia, Germany. (Nr. 23302/2020/81). Our study conforms with the World Medical Association Declaration of Helsinki.

Demographic data, laboratory parameters, mRS (modified Rankin Scale) (van Swieten et al., 1988) at admission and discharge, ICU admission, as well as mortality rates were collected. Comorbidities and, in addition, preexisting CNDs were described. These included history of stroke, epilepsy, Parkinson's disease and diseases with cognitive impairment (summarized as dementia), as well as multiple sclerosis and myasthenia (see Section 3).

### 2.2 Outcomes

For each COVID‐19 patient, data on various complications were collected. This included nonneurologic complications, such as acute respiratory distress syndrome (ARDS) and sepsis (for complete list, see Tables 2 and 4). Additionally, neurological complications were categorized into acute cerebrovascular disease, epileptic seizures, encephalopathy, and others. Cerebrovascular complications included ischemic stroke, transient ischemic attack (TIA), hypoxic brain damage, intracerebral hemorrhage, subarachnoid hemorrhage, and focal neurological deficit without correlation in brain imaging. Encephalopathy was defined as a temporary change in the mental status and consciousness. Delirium was diagnose if additional aspects such as sleep‐wake disturbance, mental and alertness fluctuation and cognitive disturbance were present. Documentation was screened also for disturbance of consciousness, signs of self‐harm or harm of others, and necessity of treatment with medication. Other complications included CIP/CIM (critical illness polyneuropathy/myopathy), tremor, ataxia, depression, severe headache, and isolated neuropathy. Nonspecific neurological symptoms such as fatigue, mild/moderate headaches, myalgia, dysgeusia, and anosmia were not considered as neurological complications.

In our first analysis, patients were divided into two groups according to the presence of a CND in comparison to patients without. For our second independent analysis, all patients were screened for the presence of ANCs in comparison to patients without. In clinical routine, patients were consulted by a neurologist as soon as they were symptomatic.

Outcome variables for regression analyses were survival and the dichotomized mRS at discharge. A favorable outcome was defined as mRS 0–3 at discharge, varying from asymptomatic to moderate disability requiring partial assistance but able to walk unassisted.

### 2.3 Statistical analysis

Descriptive statistics (median, interquartile range, and absolute and relative frequencies) were applied to compare groups. The rate of missing data was 0.07%, so imputations techniques for missing data were not applied. For pairwise comparisons, we used hypothesis testing. Nominal variables were compared using chi‐square test for independence or Fisher's exact test. The Mann–Whitney *U* test was used for appropriate occasions with ordinal or metric variables. The significance level was Bonferroni corrected relative to the amount of subgroup analyses (demographics, comorbidities, complications, and further outcomes) that were done.

Multiple logistic regression models for outcome variables were used. The regression models were adjusted for sex, age, and other risk factors such as hypertension, renal insufficiency, chronic obstructive pulmonary disease (COPD), diabetes mellitus, and coronary artery disease. For mRS at discharge, we added a baseline adjustment, namely, the prehospitalization mRS. A *p*‐value of <.05 was considered statistically significant. Odds ratios (ORs) and their 95% confidence intervals (CIs) were reported. Sample size calculations were not performed. Data analysis was conducted with IBM SPSS Statistics 27 and the statistical language R (4.0.2) (R Core Team, 2020).

## 3 RESULTS

### 3.1 Study population and clinical characteristics

Tables 1 and 2 show data of the study population. In total, 709 patients admitted to the hospital with confirmed SARS‐CoV2 infection were included in this analysis. Among them, 52.6% (*n* = 373) were males and 47.4% (*n* = 336) were females. The median age was 78 years (interquartile range [IQR]: 66–84; Figure S1), with 49.8% (*n* = 353) of the patients with a preexisting level of care and 114 patients (18.8%) with a preadmission mRS of 4 (severely disabled) or 5 (bedridden). Patients stayed in hospital for a median of 10 days (IQR: 6–16).

**TABLE 1:** Demographic data of all patients divided into those with and without preexisting chronic neurological disorders.

	All patients	CND	Non‐CND	*p*‐value
Demographic data				*α*_adj_ = .0071
Number (*n*)	709	250	459	–
Age—years (IQR)	78 (66–84)	82 (76–86)	74 (63–82)	**<.001**
Male—*n* (%)	373 (52.6%)	134 (53.6%)	239 (52.1%)	.753
mRS—prehospital	2	3	0	**<.001**
Median length of stay in ICU—days (IQR)	5 (3–11)	3 (2–9.5)	6 (3–12)	.042
Duration of mechanical ventilation—days (IQR)	10.5 (7–15)	12 (7–14)	9.5 (7–15)	.058
Median length of hospital stay—days (IQR)	10 (6–16)	11 (6–15)	10 (7–16)	.814
mRS—discharge	3	4	2	**<.001**

*Note*: Descriptive data are given as median (IQR—interquartile range) or absolute numbers (%). The significance level has been adapted to the number of pairwise comparisons done in this subanalysis (*α*_adj_). Remarkable *p*‐values with respect to *α*_adj_ are in bold numbers. Modified Rankin scale (mRS) is defined as mRS 0—no symptoms; 1—symptoms without disability; 2—unable to carry out all previous activities; 3—moderate disability, still able to walk; 4—unable to walk unassisted; 5—bedridden; and 6—dead.

Abbreviations: CND, chronic neurological disease; ICU, intensive care unit; non‐CND, no chronic neurological disease.

**TABLE 2:** Comorbidities, complications, and hospital outcomes of all patients divided into those with and without preexisting chronic neurological disorders.

	All patients	CND	Non‐CND	OR [LCI, UCI]	*p*‐value
Comorbidities					*α*_adj_ = .0056
Hypertension	600 (84.6%)	231 (92.4%)	369 (80.4%)	**2.96 [1.41, 6.21]**	**<.001**
Diabetes	248 (35%)	96 (38.4%)	152 (33.1%)	1.26 [0.80, 1.99]	.162
Heart failure	178 (25.1%)	79 (31.6%)	99 (21.6%)	**1.68 [1.02, 2.75]**	**.004**
Coronary artery disease	120 (16.9%)	52 (20.8%)	68 (14.8%)	1.51 [0.85, 2.66]	.047
Atrial fibrillation	207 (29.2%)	89 (35.6%)	118 (25.7%)	**1.60 [0.99, 2.56]**	**.007**
COPD	73 (10.3%)	23 (9.2%)	50 (10.9%)	0.83 [0.39, 1.73]	.478
PAOD	49 (6.9%)	22 (8.8%)	27 (5.9%)	1.54 [0.67, 3.54]	.163
Tumor disease	127 (17.9%)	38 (15.2%)	89 (19.4%)	0.74 [0.41, 1.34]	.183
Renal insufficiency	190 (26.8%)	92 (36.8%)	98 (21.4%)	**2.14 [1.32, 3.48]**	**<.001**
Complications					*α*_adj_ = .0045
ARDS—*n* (%)	120 (16.9%)	46 (18.4%)	74 (16.1%)	1.17 [0.65, 2.10]	.464
Acute neurological complications—*n* (%)	117 (16.5%)	54 (21.6%)	63 (13.7%)	**1.73 [0.97, 3.08]**	**.008**
Pulmonary embolism—*n* (%)	13 (1.8%)	1 (0.4%)	12 (2.6%)	–	–
NSTEMI—*n* (%)	119 (16.8%)	58 (23.2%)	61 (13.3%)	**1.97 [1.11, 3.49]**	**<.001**
STEMI—*n* (%)	7 (1%)	5 (2%)	2 (0.4%)	–	–
Acute liver failure—*n* (%)	28 (3.9%)	8 (3.2%)	20 (4.4%)	0.73 [0.21, 2.40]	.547
Sepsis—*n* (%)	199 (28.1%)	80 (32%)	119 (25.9%)	1.35 [0.82, 2.18]	.096
Septic shock—*n* (%)	16 (2.3%)	6 (2.4%)	10 (2.2%)	1.10 [0.25, 4.79]	1
Bacteremia—*n* (%)	58 (8.2%)	19 (7.6%)	39 (8.5%)	0.89 [0.39, 2.01]	.775
Deep vein thrombosis—*n* (%)	17 (2.4%)	8 (3.2%)	9 (2%)	1.65 [0.41, 6.59]	.313
Arterial embolism—*n* (%)	5 (0.7%)	3 (1.2%)	2 (0.4%)	–	–
Urinary tract infection—*n* (%)	260 (36.7%)	109 (43.6%)	151 (32.9%)	1.58 [1.0, 2.49]	.006
Acute renal failure—*n* (%)	143 (20.2%)	61 (24.4%)	82 (17.9%)	1.48 [0.86, 2.54]	.040
Outcomes					*α*_adj_ = .01
Admission to ICU—*n* (%)	169 (23.8%)	47 (18.8%)	122 (26.6%)	0.64 [0.38, 1.07]	.021
Mechanical ventilation—*n* (%)	52 (7.3%)	12 (4.8%)	40 (8.7%)	0.53 [0.21, 1.3]	.070
NIV—*n* (%)	118 (16.6%)	28 (12,6%)	90 (24,4%)	**0.52 [0.28, 0.95]**	**.004**
Tracheostomy—*n* (%)	14 (1.97%)	2 (0.8%)	12 (2.6%)	–	–
Catecholamines—*n* (%)	65 (9.2%)	21 (8.4%)	44 (9.6%)	0.86 [0.42, 1.8]	.683
Death—*n* (%)	176 (24.8%)	96 (38.4%)	80 (17.5%)	**2.95 [1.83, 4.73]**	**<.001**
De‐escalation of therapy—*n* (%)	164 (23.1%)	95 (38%)	69 (15%)	**3.46 [2.13, 5.64]**	**<.001**

*Note*: Descriptive data are given as median (IQR—interquartile range) or absolute numbers (%). Corresponding odds ratios (OR) and confidence intervals are presented, where LCI means lower boundary and UCI means upper boundary of a confidence interval. The significance level has been individually adapted to the number of pairwise comparisons done in the related subgroups (*α*_adj_). Remarkable ratios and *p*‐values with respect to *α*_adj_ are in bold numbers.

Abbreviations: ARDS, acute respiratory defiance syndrome; CND, chronic neurological disease; COPD, chronic obstructive pulmonary disease; ICU, intensive care unit; NIV, noninvasive ventilation; non‐CND, no chronic neurological disease; NSTEMI, non‐ST segment elevation myocardial infarction; PAOD, peripheral artery occlusive disease; STEMI, ST segment elevation myocardial infarction.

Most of the patients had preexisting comorbidities. The most common were hypertension in 84.6% (*n* = 600), diabetes in 35% (*n* = 248), atrial fibrillation in 29.2% (*n* = 207), and renal insufficiency in 26.8% (*n* = 190). The main cause of hospitalization was not related to SARS‐CoV2 in 8.5% (*n* = 60) of the patients. No COVID‐19 symptoms developed in 10.9% (*n* = 77) of all patients.

Apart from the common COVID‐19 symptoms (see Tables S1 and S2), the most frequently reported complications were ARDS in 16.9% (*n* = 120), non‐ST segment elevation myocardial infarction (NSTEMI) in 16.8% (*n* = 119), sepsis in 30.3% (*n* = 215), and acute renal failure in 20.2% (*n* = 143) of the patients. During the hospital treatment, 23.8% (*n* = 169) were admitted to the ICU. A standard operating procedure was established in our hospital, in which patients older than 80 years and with severe comorbidities were not admitted to ICU. The mortality rate was 24.8% (*n* = 176). Overall 23.1% (*n* = 164) of the patients had either a DNR (do not resuscitate)/DNI (do not intubate) status from the beginning or a de‐escalation of therapy with the progress of the disease.

#### 3.1.1 Patients with preexisting CNDs

Among all COVID‐19 patients, 35% (*n* = 250) had CNDs including 172 with dementia, 99 with stroke, 35 with epilepsy, 30 with Parkinson's disease, three with myasthenia gravis, and one with multiple sclerosis (see also Table S3).

Compared to non‐CND patients, those with CNDs were significantly older (median age: 82 vs. 74 years; *p* < .001) and had a higher prehospitalization mRS (3 vs. 0; *p* < .001) (Table 1).

##### Comorbidities and complications

Table 2 shows that comorbidities such as hypertension (*p* < .001) and renal insufficiency (*p* < .001) were significantly more frequent in patients with CNDs in comparison to those without. Patients with CNDs developed ANCs more often than non‐CND patients (21.6% vs. 13.7%; *p* = .008). The most common ANCs in CND patients were delirium with known dementia (10.4%; *n* = 26) and encephalopathy (6.8%; *n* = 31). Less frequent were epileptic seizures (*n* = 8), ischemic stroke (*n* = 6), intracerebral bleeding (*n* = 2), TIA (*n* = 1), hypoxic brain damage (*n* = 1), and delirium without dementia (*n* = 1). Other neurological complications in this group were also rare (CIP/CIM: *n* = 3; depression: *n* = 1; cerebral metastasis: *n* = 1; severe headache: *n* = 1). Moreover, patients with CNDs had a higher risk to develop acute renal failure (OR = 1.48; corrected CI: 0.86–2.54) and NSTEMI (OR = 1.97; corrected CI: 1.11–3.49).

##### ICU‐related outcomes

**FIGURE 1:**
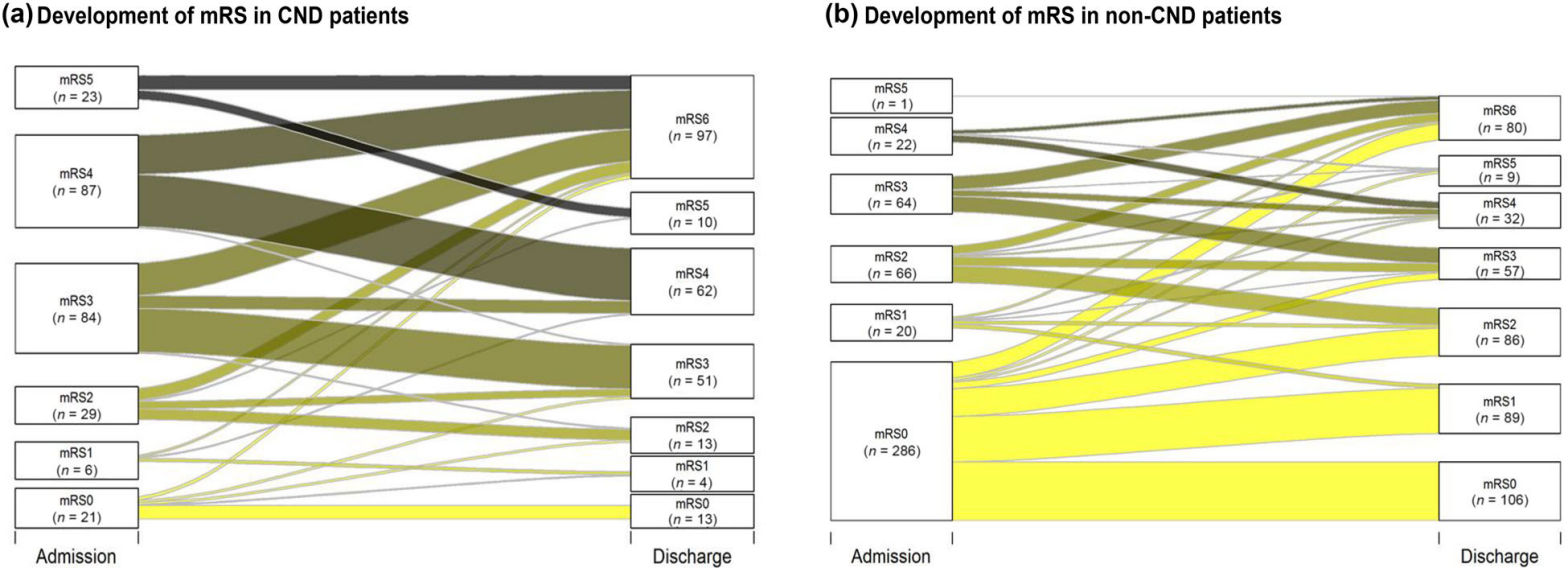
Alluvial plots demonstrating mRS at hospital admission and discharge in patients divided into two groups: CND and non‐CND. (a) Development of mRS in CND patients. (b) Development of mRS in non‐CND patients. Alluvial plots of the mRS classes for patients with preexisting neurological disorders (CND; total number: [a] 250 and [b] 459). Left: mRS classes 0–5 and their counts at the time of hospital admission. Right: mRS classes 0–6 and their counts at the time of hospital discharge. The plot reveals the number of patients crossing from one class to another after hospitalization. Dark shaded stream fields depict worse mRS status at admission time. Of all patients with mRS 0–3 (a: *n* = 140, b: *n* = 436) at admission time 42.9% (a) respectively 22.5% (b) developed an mRS 4–6. Note that box height of low group counts on both sides does not reflect the group size. All mRS groups with count of at most 32 have increased box heights. The heights are increased for illustrative reasons.

CND patients had a worse mRS score at discharge compared to non‐CND (mRS: 4 vs. 2; *p* < .001; Table 1; Figure 1) and a significantly higher mortality (38.4% vs. 17.4%; *p* < .001; Tables 1 and 2) with an almost threefold higher chance to die compared to patients without CNDs. However, patients with CNDs had a lower rate of ICU admission in comparison to non‐CND patients (18.8% vs. 26.6%; *p* = .021). They also received less frequently a noninvasive ventilation (NIV) (12.6% vs. 24.4%, respectively; *p* = .004).

Moreover, 38.2% (*n* = 271) of the CND patients versus 16.2% (*n* = 44) of the non‐CND patients had a strictly conservative therapy or de‐escalation of therapy (comfort care) (*p* < .001).

##### Prediction models

**FIGURE 2:**
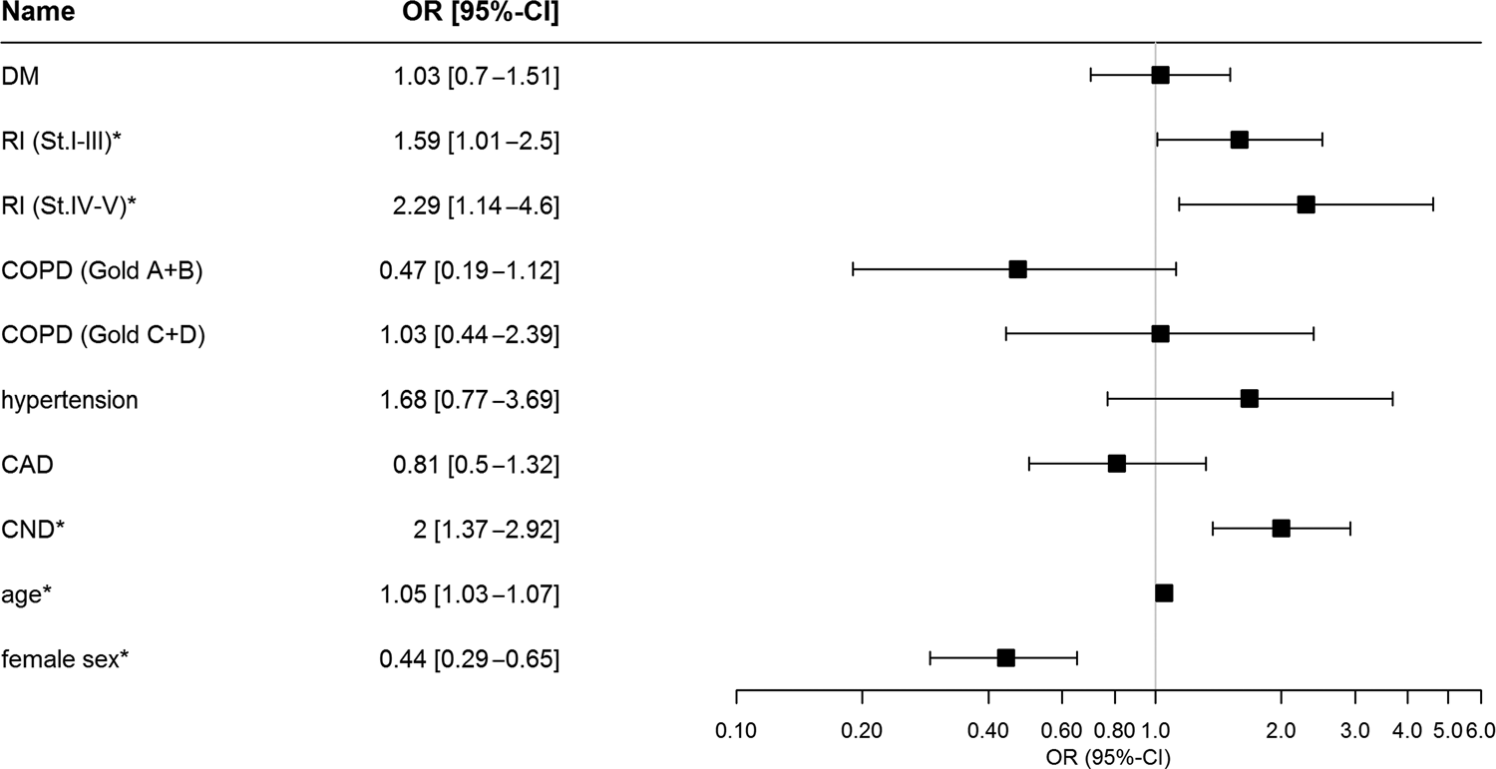
Regression model for survival with CND as predictor. Forest plot for the results of a multiple regression with survival as response. Left: List of the considered predictors variables. Middle: Odds ratio (OR) of the corresponding category of a predictor for death; opposite categories are reference category (95% confidence intervals [CIs] for the OR are presented in brackets). For age, the OR represents the change for survival in a 1‐year difference. Right: OR and 95% CI displayed, where the indifference value of 1.0 is highlighted; the axis is on a log scale. Statistical significance (*p* < .05) is marked with an asterisk. The corresponding *p*‐values of the regression for the predictors are presented in the supplements. DM, diabetes mellitus; RI, renal insufficiency Stadium I–III and IV–V; COPD, chronic obstructive pulmonary disease (Gold A + B and C + D state of disease); CAD, coronary artery disease; CND, chronic neurological disorder.

In the first multiple logistic regression analysis employing survival as primary endpoint, we found CNDs, male sex, renal failure as well as higher age as independent significant predictors of hospital mortality (Figure 2; Table S4).

Patients with CNDs had 2.0 times higher odds for death than non‐CND (*p* < .001; 95% CI: 1.3–3.0). Female patients had 60% lower odds to die than male (*p* < .001; OR = 0.44; 95% CI: 0.29–0.65). The odds for death were 1.6 times higher in patients with moderate renal insufficiency (*p* = .043; OR = 1.6; 95% CI: 1.01–2.5) and 2.3 times higher in those with severe renal insufficiency (*p* = .02; OR = 2.3; 95% CI: 1.14–4.6).

Figure S2 and Table S5 show the details of the regression analysis for our secondary endpoint, the dichotomized functional outcome. The abovementioned significant predictors for survival were also significant in this regression analysis, except for severe renal insufficiency. In addition, we observed a significantly worse outcome for patients with hypertension and a baseline mRS higher than 3.

#### 3.1.2 Patients with new‐onset ANCs

Of all patients, 117 patients developed a total of 135 ANCs. Tables 3 and 4 show the demographic data, comorbidities, complications, and hospital outcomes for the ANC patients. These had a significantly higher rate of cardiovascular risk factors, such as atrial fibrillation (*p* < .001) and hypertension (*p* = .001) compared to non‐ANC patients.

**TABLE 3:** Demographic data of patients divided into those with and without acute neurological complications.

	ANC	Non‐ANC	*p*‐value
Demographic data			*α*_adj_ = .0071
Number (*n*)	117	592	
Age—years (IQR)	79 (68–84)	78 (66–84)	.128
Male—*n* (%)	70 (59.8%)	303 (51.2%)	.105
mRS—prehospital	2	2	.171
Length of stay in ICU—days (IQR)	9.5 (4–24)	4 (2–8)	<.001
Duration of mechanical ventilation—days (IQR)	12.5 (7–17)	8 (4–11)	<.001
Length of hospital stay—days (IQR)	17 (11–26)	9 (6–15)	<.001
mRS—discharge	4	3	<.001

*Note*: Descriptive data are given as median (IQR—interquartile range) or absolute numbers (%). The significance level has been adapted to the number of pairwise comparisons done in this subanalysis (*α*_adj_). Remarkable *p*‐values with respect to *α*_adj_ are in bold numbers. Modified Rankin scale (mRS) is defined as mRS 0—no symptoms; 1—symptoms without disability; 2—unable to carry out all previous activities; 3—moderate disability, still able to walk; 4—unable to walk unassisted; 5—bedridden; and 6—dead.

Abbreviations: ANC, acute neurological complications; ICU, intensive care unit; non‐ANC, no acute neurological complication.

**TABLE 4:** Comorbidities, complications, and hospital outcomes of all patients divided into those with and without acute neurological complications.

	ANC	Non‐ANC	OR [LCI, UCI]	*p*‐value
Comorbidities				*α*_adj_ = .0056
Hypertension	110 (94%)	490 (82.8%)	**3.27 [1.06, 10.05]**	**.001**
Diabetes	45 (38.5%)	203 (34.3%)	1.20 [0.67, 2.14]	.397
Heart failure	38 (32.5%)	140 (23.6%)	1.55 [0.84, 2.86]	.048
Coronary artery disease	23 (19.7%)	97 (16.4%)	1.25 [0.61, 2.56]	.418
Atrial fibrillation	50 (42.7%)	157 (26.5%)	**2.07 [1.15, 3.69]**	**<.001**
COPD	15 (12.8%)	53 (9.8%)	1.35 [0.57, 3.20]	.320
PAOD	6 (5.1%)	43 (7.3%)	0.69 [0.19, 2.40]	.549
Tumor disease	20 (17.1%)	107 (18.1%)	0.93 [0.44, 1.97]	.895
Renal insufficiency	31 (26.5%)	159 (26.9%)	0.98 [0.52, 1.86]	.936
Complications				*α*_adj_ = .0045
ARDS—*n* (%)	19 (16.2%)	101 (17.1%)	0.94 [0.44, 2.02]	.893
Pulmonary embolism—*n* (%)	3 (2.6%)	10 (1.7%)	–	–
NSTEMI—*n* (%)	34 (29.1%)	85 (14.4%)	**2.44 [1.26, 4.69]**	**<.001**
STEMI—*n* (%)	1 (0.9%)	6 (1%)	–	–
Acute liver failure—*n* (%)	14 (12%)	14 (2.4%)	**5.61 [1.88, 16.68]**	**<.001**
Sepsis—*n* (%)	61 (52.1%)	138 (23.3%)	**3.58 [2.0, 6.4]**	**<.001**
Septic shock—*n* (%)	5 (4.3%)	11 (1.9%)	2.36 [0.51, 10.82]	.161
Bacteremia—*n* (%)	19 (16.2%)	39 (6.6%)	**2.75 [1.19, 6.33]**	**.002**
Deep vein thrombosis—*n* (%)	4 (3.4%)	13 (2.2%)	1.58 [0.31, 7.9]	.503
Arterial embolism—*n* (%)	0	5 (0.8%)	–	–
Urinary tract infection—*n* (%)	49 (41.9%)	211 (35.6%)	1.30 [0.73, 2.31]	.209
Acute renal failure—*n* (%)	45 (38.5%)	98 (16.6%)	**3.15 [1.71, 5.8]**	**<.001**
Outcomes				*α*_adj_ = .01
Admission to ICU—*n* (%)	60 (51.3%)	109 (18.4%)	**4.66 [2.65, 8.19]**	**<.001**
Mechanical ventilation—*n* (%)	28 (23.9%)	24 (4.1%)	**7.45 [3.37, 16.46]**	**<.001**
NIV—*n* (%)	42 (35.9%)	76 (12.8%)	**3.80 [2.08, 6.95]**	**<.001**
Tracheostomy—*n* (%)	11 (9.4%)	3 (0.5%)	–	–
Catecholamines—*n* (%)	30 (25.6%)	35 (5.9%)	**5.49 [2.66, 11.32]**	**<.001**
Death—*n* (%)	43 (36.8%)	133 (22.5%)	**2.00 [1.13, 3.54]**	**.002**
De‐escalation of therapy—*n* (%)	38 (32.5%)	126 (21.3%)	1.78 [0.99, 3.2]	.012

*Note*: Descriptive data are given as median (IQR—interquartile range) or *n* (%). Corresponding odds ratios (OR) and confidence intervals are presented, where LCI means lower boundary and UCI means upper boundary of a confidence interval. The significance level has been individually adapted to the number of pairwise comparisons done in the related subgroups (*α*_adj_). Remarkable ratios and *p*‐values are in bold numbers. In 117 individual patients, 135 acute neurological complications (ANCs) occurred. For our comparison, we only refer to independent data; multiple ANCs are not further considered.

Abbreviations: ANC, acute neurological complications; ARDS, acute respiratory defiance syndrome; COPD, chronic obstructive pulmonary disease; ICU, intensive care unit; NIV, noninvasive ventilation; non‐ANC, no acute neurological complication; NSTEMI, non‐ST segment elevation myocardial infarction; PAOD, peripheral artery occlusive disease; STEMI, ST segment elevation myocardial infarction.

##### Comorbidities and complications

Table 4 shows that patients with ANCs had a more complicated clinical course compared to patients without ANCs. They were longer hospitalized (median: 17 vs. 9 days; *p* < .001). They showed higher risk for developing sepsis (52.1% vs. 23.3%; *p* < .001), acute renal failure (38.5% vs. 16.6%; *p* < .001), acute liver failure (12% vs. 2.4%; *p* < .001), and NSTEMI (29.1% vs. 14.4%; *p* < .001).

Among ANCs, cerebrovascular events such as ischemic stroke occurred in 1.3% (*n* = 9), intracerebral hemorrhage in 0.8% (*n* = 6), TIA in 0.6% (*n* = 4), and subarachnoid hemorrhage in 0.1% (*n* = 1) and one patient showed a hypoxic brain damage after cardiopulmonary resuscitation. Encephalopathic complications occurred in 11.8% (*n* = 84) patients including 5.5% (*n* = 39) with encephalopathy, 3.7% (*n* = 26) with delirium with dementia, 2.5% (*n* = 18) with delirium without dementia, and 0.1% (*n* = 1) with alcohol withdrawal with delirium tremens. Epileptic seizures occurred in 1% (*n* = 7) with the manifestation of a status epilepticus in one of them. One patient had myoclonia.

Other complications occurred in 3% (*n* = 21) with 1.7% (*n* = 12) CIP/CIM, 0.3% (*n* = 2) severe headache, 0.3% (*n* = 2) depression, 0.1% (*n* = 1) isolated neuropathy in oculomotor nerve, 0.1% (*n* = 1) benign paroxysmal positional vertigo, 0.1% (*n* = 1) alcohol withdrawal, 0.1% (*n* = 1) tremor, and 0.1% (*n* = 1) first diagnosis of cerebral metastasis.

In total, 0.85% (*n* = 6) received lumbar punctions; however, SARS‐CoV2 was not detected in the cerebrospinal fluid.

##### ICU‐related outcomes

**FIGURE 3:**
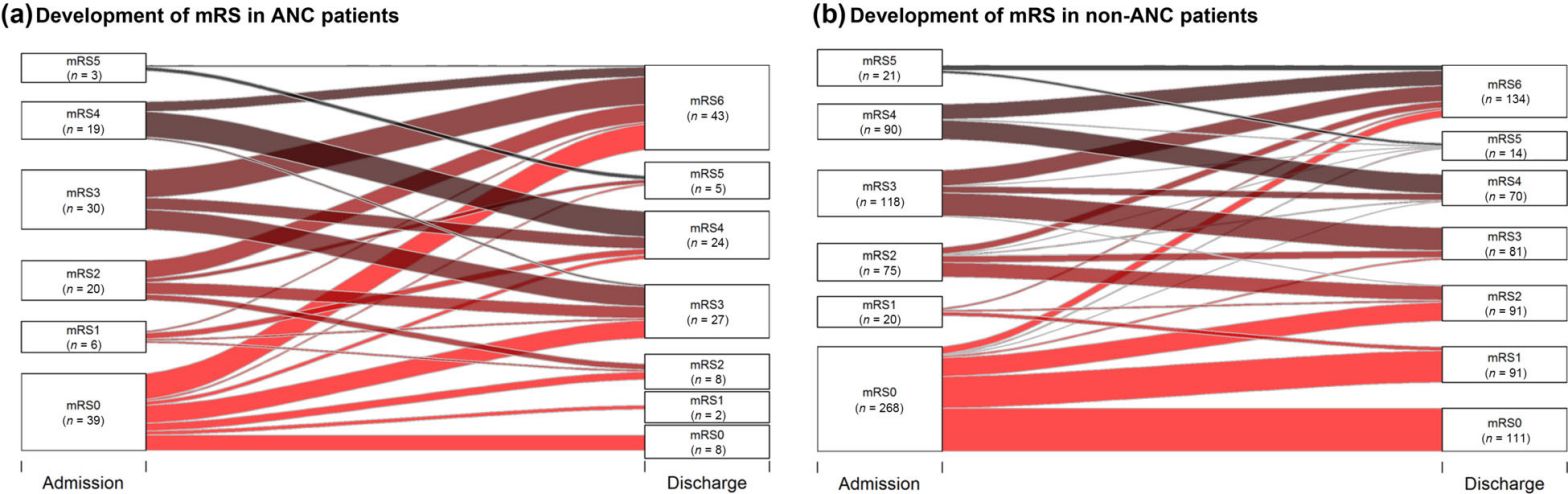
Alluvial plots demonstrating mRS at hospital admission and discharge in patients divided into two groups: ANC and non‐ANC. (a) Development of mRS in ANC patients. (b) Development of mRS in non‐ANC patients. Alluvial plots of the mRS classes for patients with acute neurological complications (ANC; total number: [a] 117 and [b] 592). Left: mRS classes 0–5 and their counts at the time of hospital admission. Right: mRS classes 0–6 and their counts at the time of hospital discharge. The plot reveals the number of patients crossing from one class to another after hospitalization. Dark shaded stream fields depict worse mRS status at admission time. Of all patients with mRS 0–3 (a: *n* = 95, b: *n* = 481) at admission time 53.7% (a) respectively 22.3% (b) developed an mRS 4–6. Note that box height of low group counts on both sides does not reflect the group size. All mRS groups with count of at most 8 (a) respectively 75 (b) have increased box heights. The heights are increased for illustrative reasons.

ANC patients had a worse mRS at discharge compared to non‐ANC patients (mRS: 3 vs. 2; *p* < .0.01; Table 3; Figure 3) and a significant higher rate of mortality (36.8% vs. 22.5%; *p* = .002).

Patients with ANCs had a higher rate of ICU admission (51.3% vs. 18.4%; *p* < .001). They received more often intubation (*p* < .001) and NIV (*p* < .001). Duration of mechanical ventilation was also significantly longer in patients with ANCs (*p* < .001). ANC patients had in total a significantly longer ICU treatment in comparison to those without ANCs (9.5 vs. 4 days; *p* < .001).

In tendency, more ANC patients (*n* = 38; 32.5%) had a strictly conservative therapy or de‐escalation of therapy (comfort care) (*p* = .012) in comparison to non‐ANC patients (*n* = 126; 21.3%), although the difference was not significant.

##### Prediction models

**FIGURE 4:**
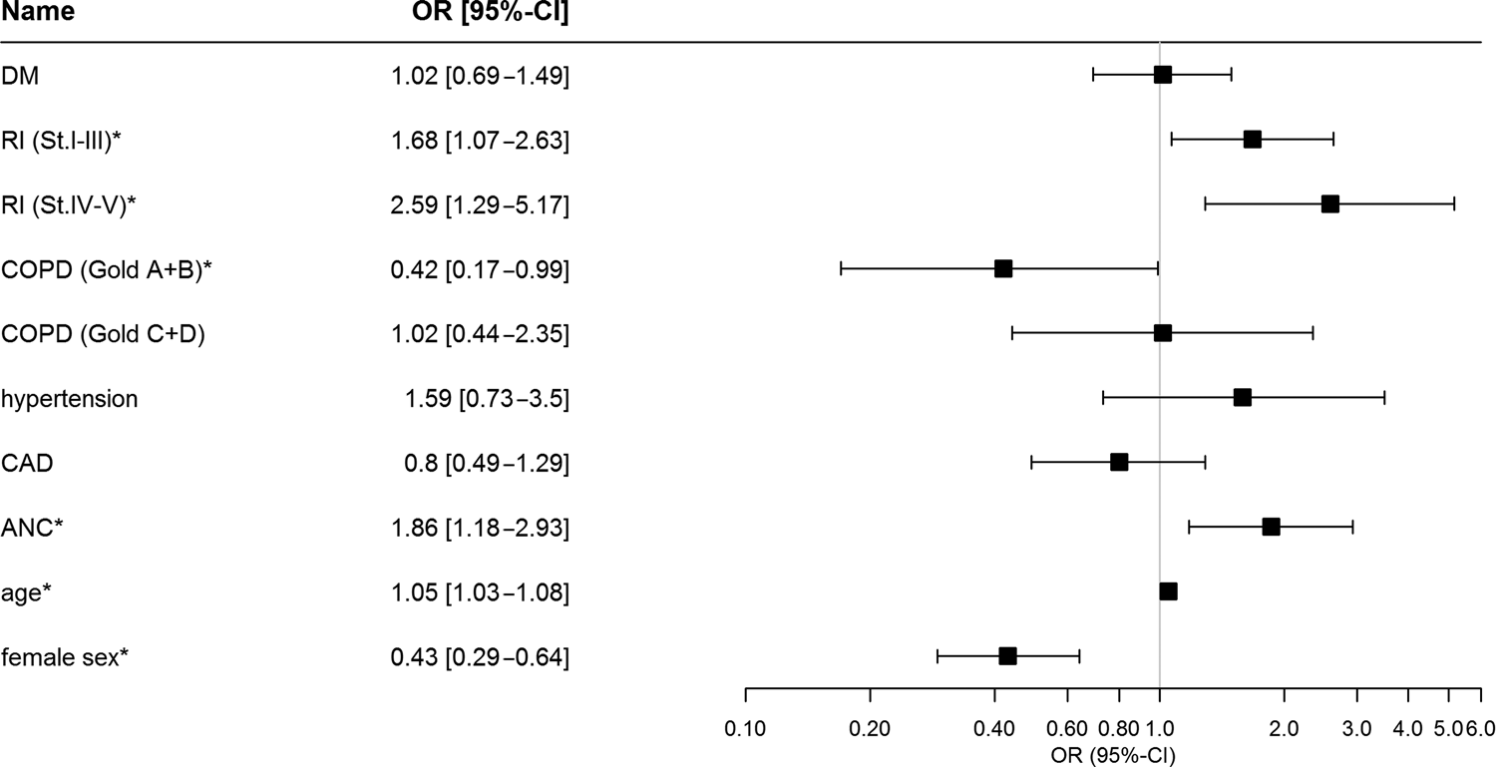
Regression model for survival with ANC as predictor. Forest plot for the results of a multiple regression with survival as response. Left: List of the considered predictors variables. Middle: Odds ratio (OR) of the corresponding category of a predictor for death; opposite categories are reference category (95% confidence intervals [CIs] for the OR are presented in brackets). For age, the OR represents the change for survival in a 1‐year difference. Right: OR and 95% CI displayed, where the indifference value of 1.0 is highlighted; the axis is on a log scale. Statistical significance (*p* < .05) is marked with an asterisk (*). The corresponding *p*‐values of the regression for the predictors are presented in the supplements. DM, diabetes mellitus; RI, renal insufficiency Stadium I–III and IV–V; COPD, chronic obstructive pulmonary disease (Gold A+B and C+D state of disease); CAD, coronary artery disease; ANC, acute neurological complication.

In the first multiple logistic regression analysis employing survival as primary endpoint, we found ANCs, male sex, moderate COPD, renal insufficiency as well as higher age as independent significant predictors of hospital mortality (Figure 4; Table S6).

ANCs increased the odds for death 1.9‐fold (*p* = .007; OR = 1.9; 95% CI: 1.18–2.93). In females, the chance to die was almost 60% lower than in male patients (*p* < .001; OR = 0.4; 95% CI: 0.29–0.64). The odds for death were 2.59 times higher in patients with severe renal insufficiency (*p* = .007; 95% CI: 1.29–5.17). Each year of life increased the odds for death by the factor of 1.046 (*p* < .001; 95% CI: 1.025–1.068).

Figure S3 and Table S7 show the details of this regression.

## 4 DISCUSSION

In this study, we could show that CNDs in hospitalized COVID‐19 patients were associated with a higher mortality and a worse functional outcome; however, they were less frequently admitted to the ICU.

Patients with CNDs such as dementia, Parkinson's disease, history of stroke, myasthenia, and multiple sclerosis showed a strong association with a fatal or more severe course of COVID‐19. Similar results were demonstrated in previous studies (Liotta et al., 2020). We further found that these patients had a higher mRS at admission and were also significantly older. This might explain the lower rate of admission to ICU and intubation. We assume that patients with a high initial mRS are often excluded from ICU treatment due to a preexisting poorer prognosis as well as the prespecified individual therapeutic limitations (i.e., DNR/DNI orders, advanced care directives). This was the case in our study population, in which 38.2% of patients with CNDs (vs. 16.2% in other patients) were either de‐escalated in therapy or limited to conservative therapy. In addition, NIV often fails in patients with dementia due to their intolerance of the oxygen supply. Moreover, patients with cognitive impairment (dementia) are shown to receive more often a palliative care than an ICU treatment (Martin‐Jimenez et al., 2020). Thus, this might explain the decreased ICU admission and the higher rate of death in people with CNDs.

In our sample, the odds for death in patients with CNDs were 2.0 times higher than in patients without CNDs. Similar results were demonstrated in previous studies (Garcia‐Azorin et al., 2020; Kleineberg et al., 2021; Pranata et al., 2020; Zhou et al., 2020). In line with Garcia‐Azorin et al. (2020), preexisting neurological disorder was a predictor for death in COVID‐19. A higher mRS (>3) at admission was also a predictor of mortality. However, most studies did concentrate on the presence of cardiovascular risk factors and not CNDs in general. Moreover, patients with CNDs were discharged with a worse mRS. Therefore, we assume that neurologic deterioration in the course of COVID‐19 infection is to be expected in these patients.

COVID‐19 patients with CNDs are shown to have higher chance to receive a strictly conservative therapy or de‐escalation of therapy (comfort care). It remains unclear whether these patients would have survived COVID‐19 if they received a full medical treatment including invasive ventilation. We need to further address this question in order to facilitate the decision‐making in this patient population regarding offering them invasive or extended therapeutic options or de‐escalation.

In this large‐scale cohort study of hospitalized COVID‐19 patients, ANCs were detected in 19% of our study population and were associated with higher rates of other severe medical complications, such as sepsis, higher mortality, and significantly worse functional outcome at discharge compared to those without ANCs. The most common neurological complications were encephalopathy, delirium, and ischemic stroke. The incidence of ANCs was 16.5% corresponding to 135 distinct disorders in 117 of 709 patients. This is in line with previous findings in the literature (Frontera, Sabadia, et al., 2021; Maury et al., 2021; Nannoni et al., 2021; Rifino et al., 2021; Travi et al., 2021). Among these complications, ischemic stroke was observed in 1.3% and intracerebral hemorrhage in 0.84% of the patients. This is also consistent with previous publications (Ellul et al., 2020; Frontera, Sabadia, et al., 2021; Rifino et al., 2021). However, we could not determine if the strokes were due to COVID‐19 and the higher coagulopathy or if they were coincidently. Previous studies showed limitations in that regard (Tsivgoulis et al., 2020).

Earlier studies reported a higher rate of hemorrhagic stroke in COVID‐19 patients associated with extracorporeal membrane oxygenation therapy (ECMO) (Kleineberg et al., 2021). In our cohort, only six patients received ECMO, in which only one of them developed intracerebral hemorrhage. Another study (Schmidbauer et al., 2022) reported a prevalence of intracranial hemorrhages in 0.85% of critically ill patients with COVID‐19. In their cohort, 20.8% of ICU patients underwent ECMO therapy. The reported prevalence of encephalopathy varies in different studies between 7% (Ellul et al., 2020) and 31% (Liotta et al., 2020). One study examining COVID‐19 patients in ICU reported delirium in 84.3% (Helms et al., 2020); however, our results showed 11.8%. One possible reason for these discrepancies might be the lack of reporting of altered mental state by doctors without a neurological background and differences in preferred definitions and established scoring procedures.

Moreover, we also found a significant association between the development of ANCs and increased mortality rate in COVID‐19 patients. Others also demonstrated a similarly worse mRS at discharge in conjunction with a significantly longer stay in hospital and especially in ICU in ANC patients (Frontera, Yang, et al., 2021). Therefore, ANC seems to be an important risk factor for a delayed recovery in COVID‐19 patients.

In contrast to our findings, others reported a lower mortality rate in patients with isolated neurological symptoms (Travi et al., 2021). However, it is difficult to compare the prevalence of neurological complications between studies due to varying definitions. Some studies included dysgeusia and anosmia as well as headache and syncope as neurological complications (Travi et al., 2021), whereas others included less specific symptoms as myalgia and dizziness (Liotta et al., 2020). We deliberately only included severe complications with the necessity of further neurological assessment such as stroke, epileptic complications, and neuropathy. This might explain the higher mortality rate and less favorable outcome in our study.

Based on our results and literature findings, a thorough neurological evaluation for all hospitalized patients with SARS‐CoV2 infection is recommended. Preexisting neurological diseases or the development of ANCs might indicate a significantly higher risk for a poor or even fatal outcome. This entails not only short‐lasting goals like hospital mortality, but also long‐lasting disabilities and the necessity for rehabilitation, although this was not addressed in our analyses. The need for prophylactic measures, including immunization in CND patients and advanced care planning, should be strongly encouraged on the basis of our study.

### 4.1 Limitations

This was a monocentric retrospective study. The number of ANCs may be underrepresented because imaging was not regularly performed in patients who did not show obvious focal neurological deficits. Furthermore, patients in our sample with preexisting neurological disorders were significantly older than the non‐CND group and had higher prevalence in cardiovascular risk factors. This might have led to a confounding bias due to co‐existing age‐related diseases. Moreover, we did not adapt the type I error for the multiple testing procedure; hence, some of the significant results may be false positive findings. This study could not consider the effects of the type of COVID‐19 variants nor the effect of vaccination against the virus on mortality or functional outcome due to the dataset period.

## 5 CONCLUSION

COVID‐19 in patients with preexisting neurological conditions is associated with higher mortality and survival with worse functional outcomes at discharge, as well as a lower rate of ICU admission. ANCs are more frequent in patients with preexisting neurological conditions in COVID‐19 patients. They were also associated with a significantly worse neurological outcome, higher rate of severe complications such as sepsis and ARDS, and thus higher mortality. Early neurological evaluation appears of great importance for prognostication in the treatment course of COVID‐19 infection.

## CONFLICT OF INTEREST STATEMENT

Albrecht Günther received speaker's honoraria from Boehringer Ingelheim, Daichii Sankyo, Pfizer, Occlutech, and Ipsen as well as research grants from MERZ Pharma and IPSEN. The other authors declare no conflicts of interest.

### PEER REVIEW

The peer review history for this article is available at https://publons.com/publon/10.1002/brb3.3066.

## Supporting information


**Supplementary Fig. 1**: Age distribution of all included patients
**Supplementary Table 1**: Prevalence of COVID‐19 symptoms in all patients
**Supplementary Table 2**: Prevalence of neurological symptoms in all patients
**Supplementary Table 3**: Subgroups of patients with pre‐existing chronic neurological diseases (CND)
**Supplementary Table 4**: Predictors of mortality—multiple logistic regression with CND as predictor
**Supplementary Fig. 2**: Regression model for worse functional outcome with CND as predictor
**Supplementary Table 5**: Predictors of worse neurological condition at discharge (mRS > 3)—multiple logistic regression with CND as predictor
**Supplementary Table 6**: Predictors of mortality—multiple logistic regression with ANC as predictor
**Supplementary Fig. 3**: Regression model for worse functional outcome with ANC as predictor
**Supplementary Table 7**: Predictors of worse neurological condition at discharge (mRS > 3)—multiple logistic regression with ANC as predictor

## Data Availability

The data that support the findings of this study are available from the corresponding author upon reasonable request.
