# Supplementary material for: Impact of new‐onset and preexisting neurological disorders in COVID‐19 patients
Source: Brain Behav. 2023 May 18;13(7):e3066. doi: 10.1002/brb3.3066 (PMC10338781; doi:10.1002/brb3.3066)
Supplement: Supplementary file 1 — Supplementary Fig. 1: Age distribution of all included patients Supplementary Table 1: Prevalence of COVID‐19 symptoms in all patients Supplementary Table 2: Prevalence of neurological symptoms in all patients Supplementary Table 3: Subgroups of patients with pre‐existing chronic neurological diseases (CND) Supplementary Table 4: Predictors of mortality—multiple logistic regression with CND as predictor Supplementary Fig. 2: Regression model for worse functional outcome with CND as predictor Supplementary Table 5: Predictors of worse neurological condition at discharge (mRS > 3)—multiple logistic regression with CND as predictor Supplementary Table 6: Predictors of mortality—multiple logistic regression with ANC as predictor Supplementary Fig. 3: Regression model for worse functional outcome with ANC as predictor Supplementary Table 7: Predictors of worse neurological condition at discharge (mRS > 3)—multiple logistic regression with ANC as predictor [file BRB3-13-e3066-s001.docx]

# SUPPLEMENTARY MATERIAL

**Supplementary Fig. 1:** Age distribution of all included patients

**Supplementary Table 1:** Prevalence of COVID-19 symptoms in all patients

**Supplementary Table 2:** Prevalence of neurological symptoms in all patients

**Supplementary Table 3:** Subgroups of patients with pre-existing chronic neurological diseases (CND)

**Supplementary Table 4:** Predictors of mortality - multiple logistic regression with CND as predictor

**Supplementary Fig. 2:** Regression model for worse functional outcome with CND as predictor

**Supplementary Table 5:** Predictors of worse neurological condition at discharge (mRS>3) - multiple logistic regression with CND as predictor

**Supplementary Table 6:** Predictors of mortality - multiple logistic regression with ANC as predictor

**Supplementary Fig. 3:** Regression model for worse functional outcome with ANC as predictor

**Supplementary Table 7:** Predictors of worse neurological condition at discharge (mRS>3) - multiple logistic regression with ANC as predictor

**Supplementary Fig. 1: Age distribution of all included patients**

**
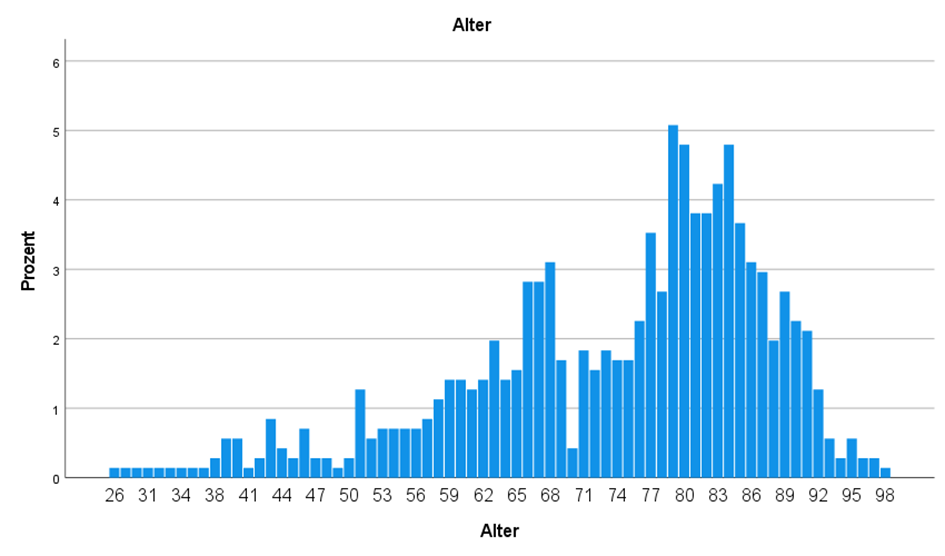
**

Relative frequency (%)

Age (years)

x-axis: age of all included patients; y-axis: relative frequency in percent

**Supplementary Table 1: Prevalence of COVID-19 symptoms** **in all patients**

| **Symptom** | **Patients** |
| --- | --- |
| fever | 64% (n=454) |
| dyspnea | 71.5% (n=507) |
| cough | 51.5% (n=365) |
| sore throat | 3.5% (n=25) |
| hypoxia | 52.9% (n=375) |
| diarrhea | 18.1% (n=128) |
| exanthema | 0,7% (n=5) |
| hemoptysis | 1.8% (n=13) |

**Supplementary Table 2: Prevalence of neurological symptoms** **in all patients**

| **Neurological symptom** | **Patients** |
| --- | --- |
| fatigue | 48.9% (n=347) |
| olfactory disorder | 6.1% (n=43) |
| dysgeusia | 10.2% (n=72) |
| headache | 6.8% (n=48) |
| myalgia | 13.3% (n=94) |

**Supplementary Table 3: Subgroups of chronic neurological diseases in CND-patients**

| **Neurological disease** | **Patients n (%)** |
| --- | --- |
| Parkinson´s Disease | 30 (4.2%) |
| **Dementia/ cognitive impairment:** | 172 (24,3%) |
| Alzheimer´s dementia | 9 (1.3%) |
| Parkinson´s disease dementia | 19 (2.7%) |
| frontotemporal dementia | 1 (0.1%) |
| vascular dementia | 17 (2.4%) |
| undefined dementia | 104 (14.7%) |
| infantile cerebral palsy | 16 (2.3%) |
| Hypoxic brain damage | 1 (0.1%) |
| Normal pressure hydrocephalus | 2 (0.3%) |
| Trisomy 21 | 2 (0.3%) |
| Multiple system atrophy | 1 (0.1%) |
| **Multiple sclerosis** | 1 (0.1%) |
| **Epilepsy** | 35 (4.9%) |
| **Myasthenia gravis** | 3 (0.4%) |
| **Cerebrovascular diseases:** | 99 (13.9%) |
| Undefined | 24 (3.4%) |
| Embolic | 36 (5.1%) |
| Microvascular | 10 (1.4%) |
| Carotid stenosis | 6 (0.8%) |
| Intracerebral hemorrhage | 10 (1.4%) |
| Multiple strokes | 7 (1%) |

**Supplementary Table 4: predictors of mortality - multiple logistic regression with CND as predictor**

| **Factor** | **OR** | **Lower 95%-CI** | **Upper 95%-CI** | **p-value** |
| --- | --- | --- | --- | --- |
| female | 0.44 | 0.29 | 0.65 | **<0.001** |
| hypertension | 1.68 | 0.77 | 3.69 | 0.195 |
| CAD | 0.81 | 0.5 | 1.32 | 0.405 |
| COPD GOLD C/D | 1.03 | 0.45 | 2.39 | 0.943 |
| COPD GOLD A/B | 0.47 | 0.19 | 1.13 | 0.090 |
| RI Stad. IV/V | 2.29 | 1.14 | 4.6 | **0.020** |
| RI Stad. I-III | 1.59 | 1.01 | 2.5 | 0.043 |
| diabetes | 1.03 | 0.7 | 1.51 | 0.886 |
| age | 1.05 | 1.03 | 1.07 | **<0.001** |
| CND | 2 | 1.37 | 2.92 | **<0.001** |

Multiple logistic regression adjusted for several variables to compare survival for patients with and without acute neurological complications; primary endpoint is death by all causes; odds ratio (OR) are listed together with its lower and upper 95% confidence interval boundaries (CI); statistical significance p<0.05 is marked **bold**; CAD coronary artery disease; COPD chronic obstructive pulmonary disease divided in moderate (GOLD stadium I and II) and severe disease (GOLD stadium III and IV). RI renal insufficiency divided in moderate (stadium I to III) and severe disease (stadium IV and V), CND chronic neurological disorders.

**Supplementary Fig. 2: regression model for worse functional outcome with CND as predictor**


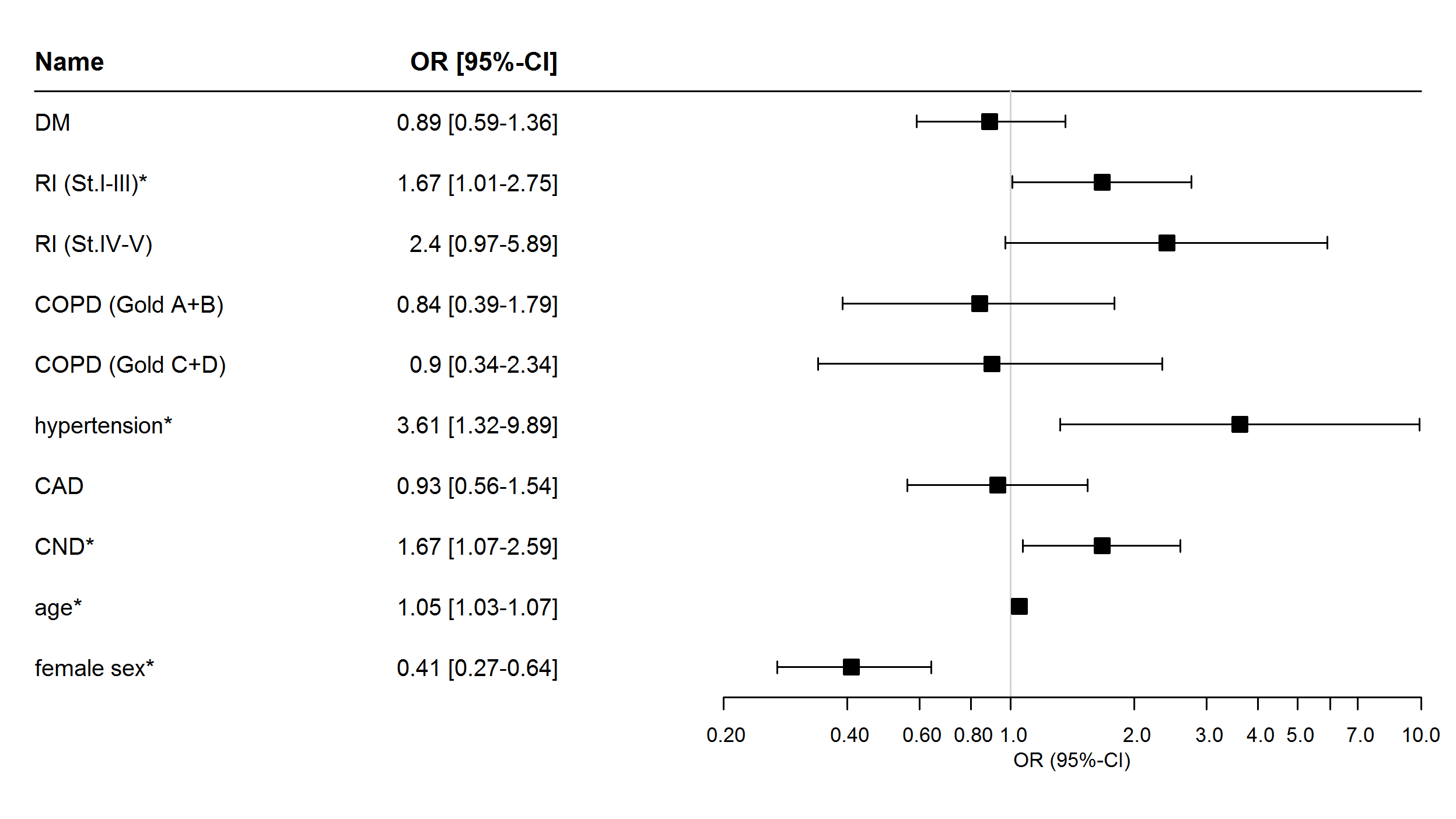


Forest plot for the results of a multiple regression with functional outcome as response. Left: list of all considered predictors variables except for the dichotomized ‘mRS before admission’, which was removed for illustrative reasons, its statistic measures can be red from Table 5; Middle: OR=Odds Ratio of the corresponding category of a predictor for a worse functional outcome, opposite categories are reference category. 95% confidence intervals (=95%-CI) for the OR are presented in brackets. For age, the OR represents the change for survival in a one-year difference. Right: OR and 95%-CI displayed, were the indifference value of 1.0 is highlighted; the axis is on a log-scale. Statistical significance (p<0.05) is marked *. The corresponding p-values of the regression for the predictors are presented in the supplements. DM diabetes mellitus, RI renal insufficiency Stadium I-III and IV-V, COPD chronic obstructive pulmonary disease (Gold A+B and C+D state of disease), CAD coronary artery disease, CND chronic neurological disorder

**Supplementary Table 5: predictors of worse functional outcome at discharge (mRS>3) - multiple logistic regression with CND as predictor**

| **Factor** | **OR** | **Lower 95%-CI** | **Upper 95%-CI** | **p-value** |
| --- | --- | --- | --- | --- |
| female | 0.41 | 0.27 | 0.64 | **<0.001** |
| hypertension | 3.61 | 1.32 | 9.89 | **0.013** |
| CAD | 0.93 | 0.56 | 1.54 | 0.767 |
| COPD GOLD C/D | 0.90 | 0.34 | 2.34 | 0.828 |
| COPD GOLD A/B | 0.84 | 0.39 | 1.79 | 0.644 |
| RI Stad. IV/V | 2.40 | 0.97 | 5.89 | 0.057 |
| RI Stad. I-III | 1.67 | 1.01 | 2.75 | **0.045** |
| diabetes | 0.89 | 0.59 | 1.36 | 0.596 |
| age | 1.05 | 1.03 | 1.07 | **<0.001** |
| mRS>3 before admission | 358 | 46 | 2781 | **<0.001** |
| CND | 1.67 | 1.07 | 2.59 | **<0.001** |

Multiple logistic regression adjusted for several variables to compare the dichotomized mRS for patients with and without pre-existing chronic neurological disorders (CND); primary endpoint is the dichotomized mRS with scores of 4 to 6 at discharge meaning inability to walk without help (mRS 4), bedridden (mRS 5) and death (mRS 6) as target event; odds ratio (OR) are listed together with its lower and upper 95% confidence interval boundaries (CI); statistical significance p<0.05 is marked **bold**; CAD coronary artery disease; COPD chronic obstructive pulmonary disease divided in moderate (GOLD stadium I and II) and severe disease (GOLD stadium III and IV). RI renal insufficiency divided in moderate (stadium I to III) and severe disease (stadium IV and V), CND chronic neurological disorders.

**Supplementary Table 6: predictors of mortality - multiple logistic regression with ANC as predictor**

| **Factor** | **OR** | **Lower 95%-CI** | **Upper 95%-CI** | **p-value** |
| --- | --- | --- | --- | --- |
| female | 0.43 | 0.29 | 0.65 | **<0.001** |
| hypertension | 1.59 | 0.73 | 3.5 | 0.244 |
| CAD | 0.80 | 0.49 | 1.30 | 0.364 |
| COPD GOLD C/D | 1.02 | 0.43 | 2.36 | 0.967 |
| COPD GOLD A/B | 0.42 | 0.17 | 0.99 | **0.047** |
| RI Stad. IV/V | 2.59 | 1.29 | 5.17 | **0.007** |
| RI Stad. I-III | 1.68 | 1.07 | 2.63 | **0.024** |
| diabetes | 1.02 | 0.69 | 1.49 | 0.936 |
| age | 1.06 | 1.03 | 1.08 | **<0.001** |
| ANC | 1.86 | 1.18 | 2.93 | **0.007** |

Multiple logistic regression adjusted for several variables to compare survival for patients with and without acute neurological complications; primary endpoint is death by all causes; odds ratio (OR) are listed together with its lower and upper 95% confidence interval boundaries (CI); statistical significance p<0.05 is marked **bold**; CAD coronary artery disease; COPD chronic obstructive pulmonary disease divided in moderate (GOLD stadium I and II) and severe disease (GOLD stadium III and IV). RI renal insufficiency divided in moderate (stadium I to III) and severe disease (stadium IV and V), ANC acute neurological complications.

**Supplementary Fig. 3: regression model for worse functional outcome with ANC as predictor**


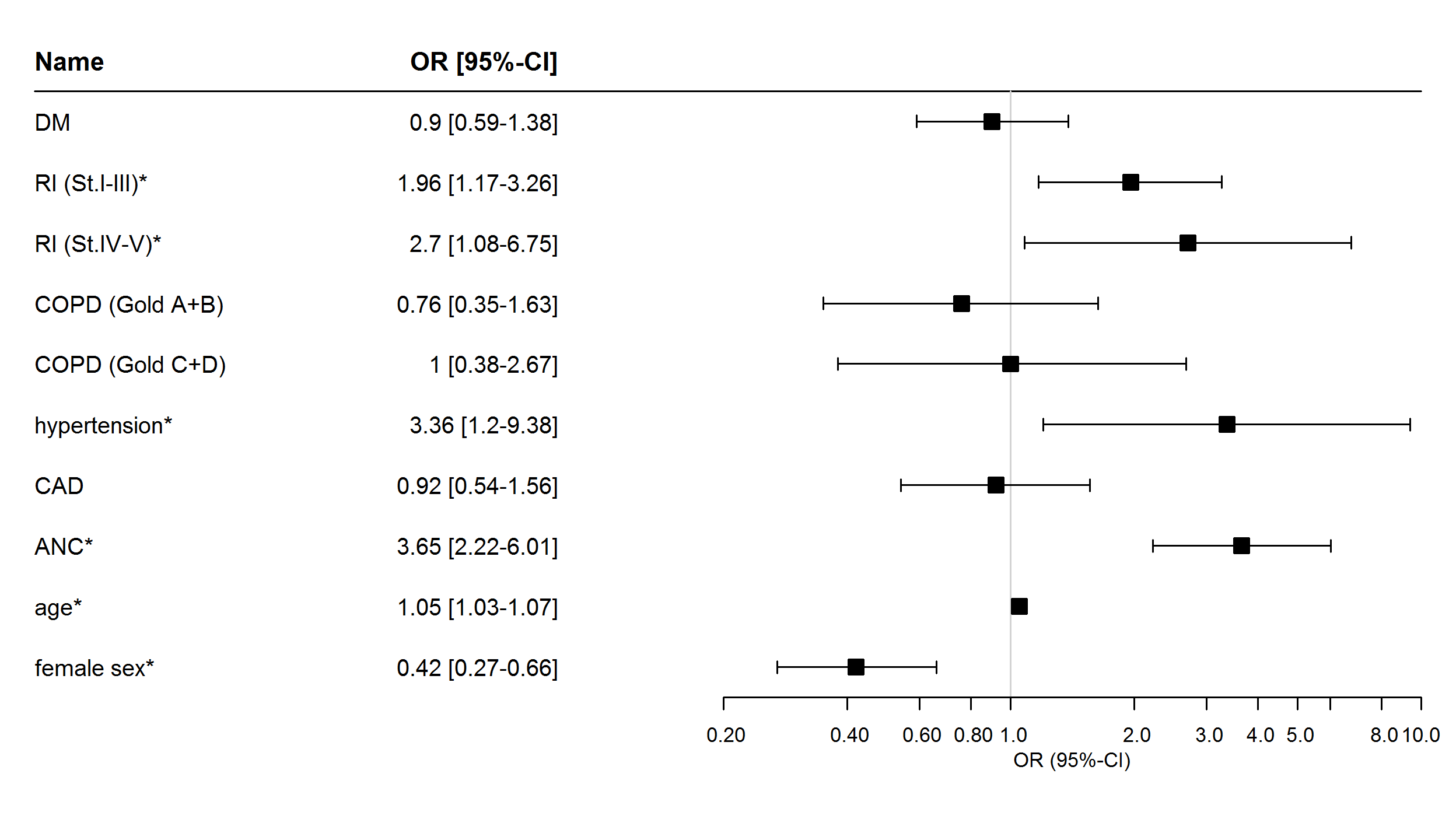


Forest plot for the results of a multiple regression with functional outcome as response. Left: list of all considered predictors variables except for the dichotomized ‘mRS before admission’, which was removed for illustrative reasons, its statistic measures can be red from Table 6; Middle: OR=odds ratio of the corresponding category of a predictor for a worse functional outcome, opposite categories are reference category. 95% confidence intervals (=95%-CI) for the OR are presented in brackets. For age, the OR represents the change for survival in a one-year difference. Right: OR and 95%-CI displayed, were the indifference value of 1.0 is highlighted; the axis is on a log-scale. Statistical significance (p<0.05) is marked *. The corresponding p-values of the regression for the predictors are presented in the supplements. DM diabetes mellitus, RI renal insufficiency Stadium I-III and IV-V, COPD chronic obstructive pulmonary disease (Gold A+B and C+D state of disease), CAD coronary artery disease, ANC acute neurological complications

**Supplementary Table 7: predictors of worse functional outcome at discharge (mRS>3) - Multiple logistic regression with ANC as predictor**

| **Factor** | **OR** | **Lower 95%-CI** | **Upper 95%-CI** | **p-value** |
| --- | --- | --- | --- | --- |
| female | 0.42 | 0.27 | 0.66 | **<0.001** |
| hypertension | 3.36 | 1.2 | 9.38 | **0.021** |
| CAD | 0.92 | 0.54 | 1.56 | 0.757 |
| COPD GOLD C/D | 1 | 0.38 | 2.67 | 0.998 |
| COPD GOLD A/B | 0.76 | 0.35 | 1.63 | 0.476 |
| RI Stad. IV/V | 2.7 | 1.08 | 6.75 | **0.034** |
| RI Stad. I-III | 1.96 | 1.17 | 3.26 | **0.010** |
| diabetes | 0.9 | 0.59 | 1.38 | 0.637 |
| age | 1.05 | 1.03 | 1.07 | **<0.001** |
| mRS>3 before admission | 526 | 68 | 4060 | **<0.001** |
| ANC | 3.65 | 2.22 | 6.01 | **<0.001** |

Multiple logistic regression adjusted for several variables to compare the dichotomized mRS for patients with and without acute neurological complications; primary endpoint is the dichotomized mRS with scores of 4 to 6 at discharge meaning inability to walk without help (mRS 4), bedridden (mRS 5) and death (mRS 6) as target event; odds ratio (OR) are listed together with its lower and upper 95% confidence interval boundaries (CI); statistical significance p<0.05 is marked **bold**; CAD coronary artery disease; COPD chronic obstructive pulmonary disease divided in moderate (GOLD stadium I and II) and severe disease (GOLD stadium III and IV). RI renal insufficiency divided in moderate (stadium I to III) and severe disease (stadium IV and V), ANC acute neurological complications.
